# Cellular Stress and Molecular Responses in Bladder Ischemia

**DOI:** 10.3390/ijms222111862

**Published:** 2021-11-01

**Authors:** Jing-Hua Yang, Han-Pil Choi, Wanting Niu, Kazem M. Azadzoi

**Affiliations:** 1 Department of Surgery, Boston University School of Medicine, Boston, MA 02118, USA; jyang@bu.edu; 2 Proteomics Laboratory, VA Boston Healthcare System, Boston, MA 02130, USA; hpchoi89@gmail.com; 3 Research Section, VA Boston Healthcare System, Boston, MA 02130, USA; Wanting.Niu@va.gov; 4 Departments of Urology and Pathology, VA Boston Healthcare System and Boston University School of Medicine, Boston, MA 02130, USA

**Keywords:** bladder ischemia, cellular stress, post-translational modifications, non-coded amino acids

## Abstract

The concept of bladder ischemia as a contributing factor to detrusor overactivity and lower urinary tract symptoms (LUTS) is evolving. Bladder ischemia as a consequence of pelvic arterial atherosclerosis was first documented in experimental models and later in elderly patients with LUTS. It was shown that early-stage moderate ischemia produces detrusor overactivity, while prolonged severe ischemia provokes changes consistent with detrusor underactivity. Recent studies imply a central role of cellular energy sensors, cellular stress sensors, and stress response molecules in bladder responses to ischemia. The cellular energy sensor adenosine monophosphate-activated protein kinase was shown to play a role in detrusor overactivity and neurodegeneration in bladder ischemia. The cellular stress sensors apoptosis signal-regulating kinase 1 and caspase-3 along with heat shock proteins were characterized as important contributing factors to smooth muscle structural modifications and apoptotic responses in bladder ischemia. Downstream pathways seem to involve hypoxia-inducible factor, transforming growth factor beta, vascular endothelial growth factor, and nerve growth factor. Molecular responses to bladder ischemia were associated with differential protein expression, the accumulation of non-coded amino acids, and post-translational modifications of contractile proteins and stress response molecules. Further insight into cellular stress responses in bladder ischemia may provide novel diagnostic and therapeutic targets against LUTS.

## 1. Introduction

Arterial atherosclerosis is a leading cause of ischemic disorders in the human body [1]. Atherosclerotic occlusive disease can develop in any artery in the body, including the arteries of the heart, brain, kidney, legs, and pelvis. Ischemia is the most common clinical manifestation of atherosclerotic occlusive disease, resulting from an imbalance between oxygen and nutrient demand and supply due to an impaired blood flow in part of the body [1]. Ischemia diminishes the distribution of oxygen, glucose, and nutrients, leading to metabolic stress and oxidative insult with an adverse impact on cellular structure and function [2]. The production of free radicals in normal cells is tightly regulated by homeostatic mechanisms. The antioxidant defense system protects tissues from oxidative injury by neutralizing excessive free radicals. Metabolic waste within tissues is normally cleared out by blood perfusion at systemic arterial pressure. The lack of perfusion in ischemia allows for the accumulation of waste products and constitutes a hospitable environment for free radicals and inflammatory responses [2]. Long-term ischemia galvanizes free radical activity and inspires oxidative insult to vital biomolecules and cellular structures [2]. Oxidative incursion is fundamentally haphazard and overwhelming with the propensity of provoking cell danger signaling and degenerative responses [2]. These changes compromise the structural integrity of smooth muscle cells, cellular organelles, microvasculature, and nerve fibers, leading to dysfunction [2].

The clinical manifestations of systemic atherosclerosis include coronary artery disease, cerebrovascular disease, ischemic nephropathy, intestinal ischemic syndrome, arteriogenic erectile dysfunction, and chronic pelvic ischemia [3]. Compelling evidence from basic and clinical research suggests that bladder ischemia may contribute to detrusor overactivity and the development of lower urinary tract symptoms (LUTS) in both genders [4,5,6,7,8,9,10,11]. Bladder ischemia can result from aging-associated arterial insufficiency caused by atherosclerotic occlusive disease of the pelvic vessels. Basic experimental research using animal models suggests that moderate ischemia sensitizes bladder smooth muscle cells to contractile stimuli and leads to overactive bladder contractions, non-compliance, and urinary frequency [4,5,6]. In contrast, prolonged severe ischemia impairs bladder contractile activity and provokes degenerative changes in smooth muscle cells, microvasculature, and nerve fibers [7]. Functional changes with moderate and severe bladder ischemia were consistent with detrusor overactivity and underactivity, respectively [4,5,6,7]. Persisting ischemic insult in the bladder is associated with impairment of the cellular antioxidant defense system, mitochondrial structural damage, the depression of mitochondrial respiration, and the activation of cell survival signaling [8].

Metabolic stress, along with hypoxia, oxidative stress, and the accumulation of metabolic waste in bladder ischemia, disrupts cellular homeostasis and initiates defensive responses to rebalance energy homeostasis and promote survival [4,5,6,7,8]. When ischemia persists, cellular defensive capacity declines leading to cell survival signaling and stress responses [4,5,6,7,8]. Ischemia is confronted by a series of cellular and molecular responses involving the translocation of transcription factors, derangement of cellular organelles, activation of energy sensors, and upregulation of stress response molecules. Metabolic stress and cell danger signals in ischemia initiate coordinated rejoinders involving smooth muscle cells, cellular organelles, microvasculature, nerve fibers, and receptors to develop protective measures against ischemic insult. These defensive responses were shown to provoke hypersensitivity of neural and smooth muscle cells by influencing neurotransmission discharge and receptor expression, resulting in overactive smooth muscle contractions [4,5,6,7,8]. When ischemic insult continues, cellular defensive mechanisms fail and subcellular elements deteriorate leading to cell danger signals and the activation of inflammatory and degenerative responses [4,5,6,7,8].

Bladder responses to ischemia are thought to provoke LUTS, a constellation of bothersome voiding symptoms affecting both men and women as they age [9,10,11]. LUTS include frequency, urgency, nocturia, incontinence, intermittency, slow stream, hesitancy, post-micturition dribble, and the sensation of incomplete bladder emptying. While the incidence of LUTS increases with aging, our understanding of the aging factors contributing to LUTS in the elderly is evolving. The precise mechanism of LUTS in patients with non-obstructed bladder remains largely elusive. Aging-associated local changes in the bladder predisposing to detrusor overactivity and LUTS remain largely unknown. It is suggested that bladder ischemia may be an independent factor in the development of LUTS in patients with non-obstructed bladder [10,11,12,13]. It is believed that aging-associated sex-independent mechanisms involving local changes in bladder arterial supply initiate molecular responses within the smooth muscle cells, epithelium, and nerve fibers and lead to overactive detrusor contractions [10,11,12,13]. This is consistent with reports of smooth muscle hypersensitivity and overactive contractile activity in other moderately ischemic organs, including the stomach, gut, uterus, bronchioles, urethra, and the cardiovascular system [14,15,16,17].

## 2. Clinical Evidence of Bladder Ischemia

Epidemiological data suggest that LUTS closely correlate with vascular disorders [18,19,20,21,22]. A recent study involving 36,042 patients reported that the incidence of LUTS significantly correlates with the prevalence of atherosclerotic arterial occlusive disease [22]. Other studies have shown that voiding dysfunction is more prevalent in patients with cardiovascular disease in comparison with same-age patients without cardiovascular disease [18,19,20,21,22]. The American Urological Association Symptom Score (AUASS) for LUTS was significantly worse in men with cardiovascular disorders than symptomatic patients without cardiovascular problems [19,21]. The risk factors contributing to vascular occlusive disease include smoking, alcohol use, diabetes mellitus, hypertension, high cholesterol, low physical activity, and elevated body mass index (BMI). It was shown that most cases of LUTS in patients with vascular risk factors are associated with arteriogenic erectile dysfunction (ED), suggesting pelvic atherosclerosis as a unifying mechanism of LUTS-associated ED in elderly patients [23,24,25]. Other studies reported that patients being treated for cardiovascular diseases were more likely to complain of concurrent LUTS and ED [26,27]. A retrospective cohort study of patients with LUTS revealed a high incidence of vascular risk factors, including hypertension, smoking, obesity, hyperlipidemia, and elevated blood glucose [27]. Vascular risk factors among African American men were found as independent predictors of LUTS [11]. In a survey of over 4000 men, the incidence of LUTS among men with ED was significantly greater than that among men without ED [25]. Another study documented a close relationship between the severity of both LUTS and ED and the severity of vascular disorders in the aging population [28].

The concept of pelvic ischemia and the epidemiological link between LUTS and vascular disease are supported by documentation of decreased bladder blood flow in elderly patients with LUTS. Several studies have measured pelvic ischemia and reported that low bladder blood flow significantly correlates with bladder dysfunction and LUTS [29,30,31]. Concomitant pelvic ischemia and cardiac ischemia were documented in patients with vascular risk factors [29]. Another study showed that reduced human bladder compliance significantly correlates with decreased bladder blood flow [30]. A measurement of human bladder blood flow with transrectal color Doppler ultrasonography revealed significantly lower bladder perfusion in elderly patients with LUTS in comparison with asymptomatic younger patients [31]. The degrees of bladder ischemia significantly correlated with the severity of LUTS in both men and women [31]. A significant correlation between the degrees of pelvic atherosclerosis and the severity of LUTS was also reported [32]. It was shown that arterial occlusive disease may contribute to both voiding and storage dysfunction by mechanisms involving bladder ischemia and oxidative stress [32]. In men, LUTS improvement with alpha adrenoceptor blockers was associated with a significant increase in bladder blood flow [33]. An improvement in bladder blood flow and the reversal of ischemia with dutasteride were shown to reduce LUTS in elderly patients [34]. In addition, it was reported that impairment of bladder blood vessels during surgical procedures may contribute to bladder dysfunction and LUTS [35,36]. Lower urinary tract dysfunction has been documented in most patients with reduced pelvic blood flow after aortic surgery [35,36]. Impairment of bladder blood flow has also been documented following pelvic surgery to correct bladder outlet obstruction due to prostatic hyperplasia [37,38]. Impairment of bladder blood flow following transurethral resection of the prostate has been shown to provoke persistent detrusor overactivity [39].

## 3. Oxidative Stress in Bladder Ischemia

**Figure 1:**
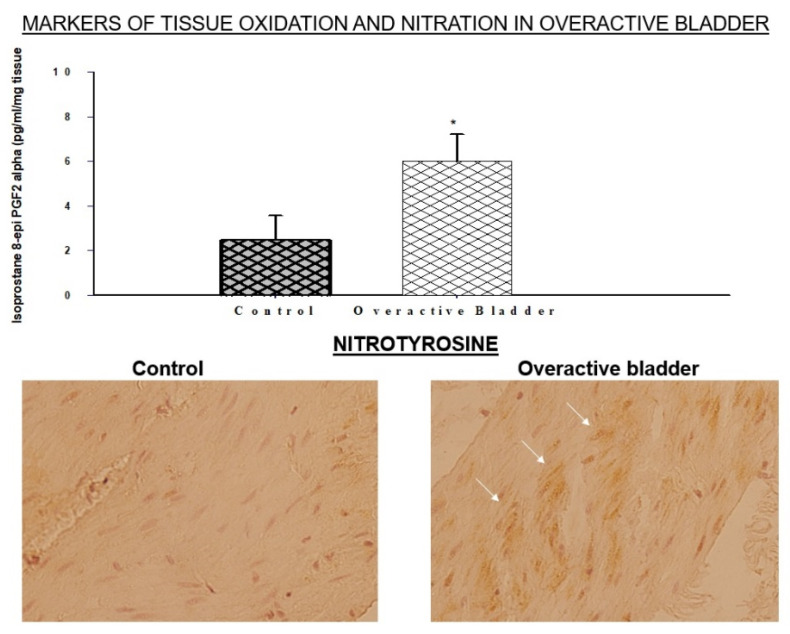
Markers of oxidative and nitrosative stress in bladder ischemia. Expression levels of the oxidative stress product isoprostane 8-epi PGF2α are upregulated in the ischemic overactive bladder as shown in the upper panel. In the lower panel, immunostaining shows diffuse nitrotyrosine immunoreactivity in the ischemic overactive bladder, suggesting nitrosative stress. * Indicates significant difference versus control. Arrows point to nitrotyrosine immunopositive areas. This figure is taken from our previous publication [40].

Muscles with persistent overactive contractions produce excessive free radicals and exhort cellular and subcellular oxidative stress [2,3]. Tissue overactivity under ischemic conditions causes a rapid decline in cellular energy resources leading to derangement of the oxidant and antioxidant defense system [2]. This results in an increased production of oxidative radicals, such as superoxide (O_2_^−^), that rapidly interact with nitric oxide (NO) and activate the nitrosative stress pathway [2,3]. The accumulation of oxidative and nitrosative radicals under ischemic conditions initiates cellular stress responses with a deleterious impact on smooth muscle cells, microvasculature, epithelium, and nerve fibers by means of DNA damage, protein oxidation, lipid peroxidation, and the upregulation of proinflammatory cytokines [2,3]. The accumulation of oxidative products and immunopositive areas of nitrotyrosine have been detected within the bladder layers, implying the activation of both oxidative and nitrosative processes (Figure 1) [40]. The O_2_^−^ and NO interaction leads to the formation of a very potent oxidant, namely peroxynitrite (O=NOO¯) [6]. The degradation of O=NOO¯ engenders cytotoxic radicals, such as nitrite and hydroxyl radicals, leading to the activation of nitrosylation reactions. O=NOO¯ initiates chain reaction byproducts with deleterious effects on cellular and subcellular components [6]. O=NOO¯ provokes rapid oxidative damage to smooth muscle cells, nerve fibers, and microvessels, and it is known to be much more potent as an oxidizing compound than hydrogen peroxide [6].

Oxidative stress contributes to the upregulation of eicosanoids and leukotrienes in the ischemic overactive bladder [4,6]. These products are known to augment bladder smooth muscle contractions, suggesting that free radicals produced by overactive contractions may exacerbate detrusor overactivity by further upregulating the formation of free radicals and constrictor eicosanoids. The production of oxidative and nitrosative radicals in normal smooth muscle cells and neurons is tightly regulated by homeostatic mechanisms. The cellular antioxidant defense system restrains free radicals and detoxifies them to prevent oxidative damage. In pathologic conditions, such as ischemia, the antioxidant defense system undergoes modifications, and, consequently, the production of free radicals exceeds the antioxidant capacity of the cells [7,8]. Excessive free radicals and enduring oxidative insult provoke degenerative responses within cellular and subcellular elements leading to cell death [7,8].

**Figure 2:**
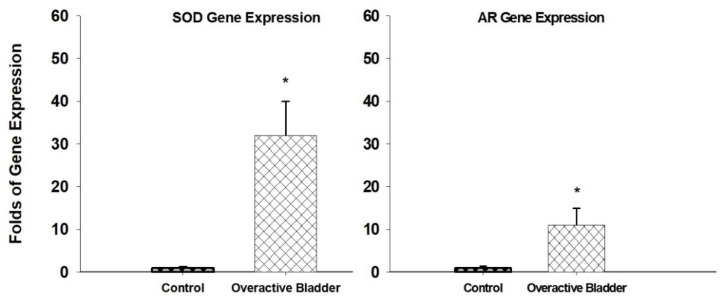
SOD and AR gene expressions are significantly upregulated in the ischemic overactive bladder tissues in comparison with controls. * Indicates significant differences versus control. This figure is taken from our previous publication [40].

The accumulation of oxidative and nitrosative radicals in the ischemic overactive bladder was shown to provoke defensive cellular responses involving oxidative stress-sensitive enzymes, such as superoxide dismutase (SOD) and aldose reductase (AR) [40]. The upregulation of both SOD and AR is documented in the ischemic overactive bladder (Figure 2) [40]. SOD diminishes peroxidation damage by catalyzing the dismutation of superoxide into oxygen and hydrogen peroxide. The systemic administration of antioxidants that mimic SOD was shown to reduce free radical activity and prevent oxidative damage in experimental models of diabetes and hypertension [40]. In contrast, the inhibition of SOD was shown to promote free radical activity and oxidative damage and obliterate the late phase of ischemic preconditioning [40]. AR acts by diminishing cytotoxic aldehydes and glutathione conjugates of aldehydes that accumulate as a consequence of lipid peroxidation [40]. AR can exhibit a dual function in stressed cells, depending on the affected organs and disease conditions. As an example, AR was shown to play a mediating role in ischemia/reperfusion injury in the heart while protecting blood vessels from free radical damage under the physiologic conditions [40]. In the ischemic bladder, the upregulation of SOD and AR did not avert degenerative responses in the mitochondria, epithelial cells, or nerve fibers, suggesting that the excessive production of free radicals overwhelms the antioxidant capacity of the cells leading to unchecked production of oxidative radicals and widespread oxidative damage [40].

## 4. Stress Response Molecules in Bladder Ischemia

Ischemia engenders metabolic stress and provokes cellular stress responses in the bladder [41,42]. Stressed cells coordinate protective responses against the ischemic insult to rebalance the compromised energy homeostasis. This activates cellular energy sensors to regulate the homeostatic process by allowing some energy to be consumed to maintain cell functionality while preserving some energy to survive the unforeseen energy deprivation consequences. Interruption of energy resources to the cells activates cellular stress response molecules and cell danger signals to prevent structural damage and promote cell function [43,44]. However, the subcellular consequences of ischemic stress depend on the severity of nutrient deficiency, the level of hypoxia, the extent of oxidative insult, and the cell’s ability to respond to the stress-eliciting rudiments [41,42,43,44]. The initial ramifications of ischemic stress involve the upregulation of stress response molecules followed by cell danger signaling and survival responses [41,42]. Three major stress response molecules have been characterized in the ischemic bladder.

**Figure 3:**
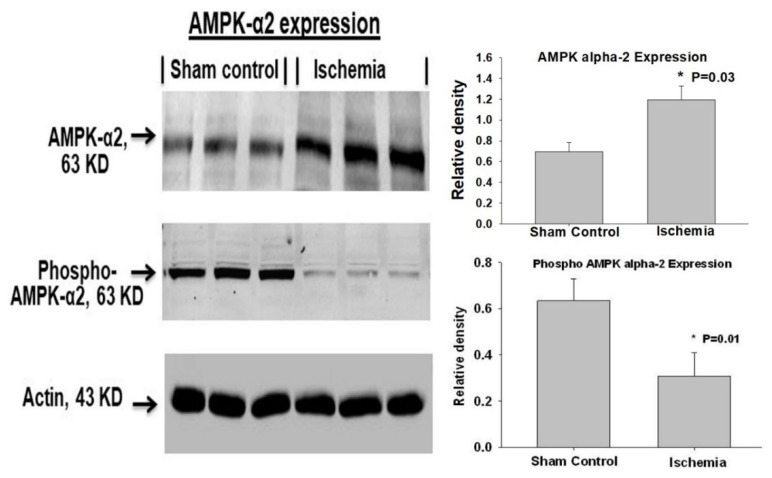
Western blotting of AMPK-α2 protein showing upregulation of total AMPK-α2 and downregulation of phosphorylated AMPK-α2 in bladder ischemia. These observations suggest that upregulated AMPK-α2 cannot be phosphorylated and remains inactive under ischemic conditions. * Represents significant changes in ischemia versus controls. This figure is taken from our previous publication [41].

(1) AMPK-α2: Adenosine monophosphate-activated protein kinase (AMPK) is an essential element of the metabolic stress sensing system. AMPK senses cellular energy status and plays a leading role in maintaining cellular energy homeostasis when energy resources decline [45,46,47]. Among the α, β, and γ subunits of AMPK, the α-2 subunit with the catalytic kinase domain (AMPK-α2) is abundantly expressed in the bladder and seems to be sensitive to ischemia [41]. It was shown that bladder ischemia upregulates total AMPK-α2 levels while downregulating the phosphorylated and, thus, activated form of AMPK-α2 (Figure 3) [41]. Impairment of AMPK-α2 appeared to augment metabolic stress in bladder ischemia, initiate cellular stress responses, and lead to structural damage and dysfunction [41].

**Figure 4:**
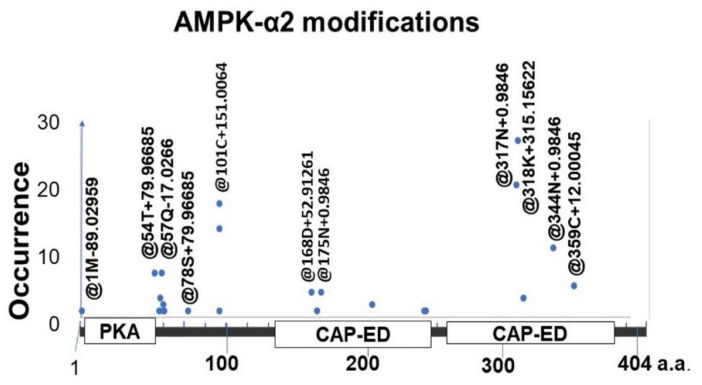
Proteomic analysis using liquid chromatography–tandem mass spectrometry (LC-MS/MS) implies post-translational modifications of AMPK-α2 protein at phosphorylation sites, including 54T + 79.967 and 78S + 79.967 near the C-terminal region of the PKA domain. Other modifications include @168D + 52.913 and @318K + 315.156 in the first and second CAP-ED motif, respectively. The modification types and positions are assigned with @ + position + amino acid + delta mass. This figure is taken from our previous publication [41].

Proteomic analysis has revealed that AMPK-α2 impairment in bladder ischemia involves post-translational modifications of its protein at multiple functional domains, including the phosphorylation sites of protein kinase A2 (Figure 4) [41].

These observations suggest that protein upregulation under ischemic conditions may not insinuate functional significance. Upregulated proteins with post-translational modifications may display a functional deficit and exhibit totally different patterns of protein–protein interaction [48,49]. Modified AMPK-α2 may not be capable of sensing declined cellular energy levels to promote homeostatic control under metabolic stress conditions in ischemia. Impairment of AMPK-α2 and subsequent disruption of cellular homeostasis in bladder ischemia may provoke DNA, RNA, and lipid damage and compromise the structural integrity of other proteins.

The functional role of AMPK-α2 in the bladder has been verified by validating the beneficial effects of the AMPK activator, 5-aminoimidazole-4-carboxamide-1-beta-D ribofuranoside (AICAR), in a rat model of bladder ischemia. AICAR is an adenosine analogue that activates AMPK by engendering allosteric modification [50]. It was shown that the treatment of rats with subcutaneous AICAR for four weeks decreases the force of overactive detrusor contractions in bladder ischemia, increases bladder capacity, and diminishes ischemic neural injury [41]. These effects of AICAR are noticeable in animals with bladder ischemia only, suggesting rebalancing of the disturbed cellular energy homeostasis as a potential mechanism. Treatment of the ischemic bladder tissues with AICAR in an organ bath diminishes contractile reactivity to electrical field stimulation [41]. The underlying mechanism of overactive smooth muscle contractions as a consequence of AMPK-α2 impairment remains largely elusive. One possibility may be the activation of cellular stress signaling and subsequent sensitization of smooth muscle cells to contractile stimuli. The role of constitutive AMPK activity in the constitutive regulation of smooth muscle contractile proteins has been documented [51]. It is suggested that AMPK activation in response to metabolic stress is required to preserve cellular energy by reducing contractile activity [51]. Vascular tissue samples from AMPK-α2 knockout mice (AMPKα2^−/−^) display greater smooth muscle contractile responses in comparison with control samples [51]. In addition, arterial blood pressure in AMPKα2^−/−^ mice was shown to be significantly greater, suggesting low arterial compliance [51]. In cultured smooth muscle cells, the inhibition of AMPK provokes the phosphorylation of both myosin light chain (MLC) and myosin phosphatase targeting subunit one (MYPT1) [51]. In contrast, AICAR was shown to inhibit the phosphorylation of MLC and MYPT1, suggesting that AMPK maintains smooth muscle contractile activity at low levels to improve compliance [51]. The protective role of AICAR against the loss of neural density in bladder ischemia suggests that AMPK may promote nerve fiber outgrowth and protect neural integrity. It was shown that AMPK regulates neuronal energy levels during synaptic activation by coordinating neural glycolysis and mitochondrial respiration [52]. The activation of AMPK by AICAR promotes the antioxidant defense system and prevents oxidative neural injury [50,51]. These observations may suggest the therapeutic potential of AMPK activators against neurodegeneration in ischemic and oxidative stress conditions.

(2) ASK1: Apoptosis signal-regulating kinase 1 (ASK1) is a cellular stress sensor that determines the fate of stressed cells by regulating downstream stress response molecules and signaling pathways [53,54,55,56]. ASK1 is activated by redox proteins following cell exposure to stressful conditions, such as hypoxia and redox [53,54]. ASK1 plays a leading role in inflammatory and degenerative responses to ischemia and oxidative stress [53,54,55,56]. Inhibition of ASK1 diminishes free radical injury and prevents structural damage [53]. ASK1 knockout mice exhibit normal homeostatic functions with significantly less structural damage after ischemia–reperfusion injury [55,56]. These observations suggest the prophylactic and therapeutic potential of ASK1 inhibitors against cellular stress, ischemic structural damage, and cell death.

**Figure 5:**
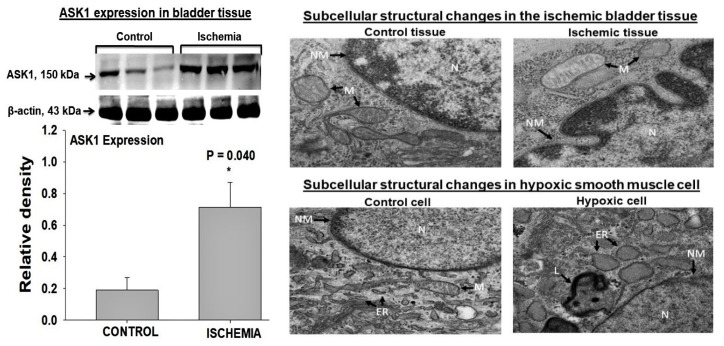
Western blotting (**left panel**) shows significant upregulation of ASK1 in ischemic rat bladder tissues [42]. Transmission electron microscopy (**right panel**) suggests widespread apoptotic activities in both ischemic bladder tissues and hypoxic smooth muscle cells involving the nucleus (N), nuclear membrane (NM), mitochondria (M), and endoplasmic reticulum (ER) and increased levels of lysosomes (L). The bladder tissue figures are reduced from 18,500×. The smooth muscle cell figures are reduced from 13,000×. This figure is taken from our previous publication [42].

ASK1 is upregulated in rat bladder ischemia, suggesting the activation of metabolic stress and redox sensing in the ischemic bladder tissue. The upregulation of ASK1 seems to provoke apoptotic activity and degenerative responses in the ischemic bladder (Figure 5) [42]. The exposure of human bladder smooth muscle cells to hypoxia upregulates ASK1 in a manner similar to that of bladder ischemia, suggesting the regulation of ASK1 expression by hypoxia under ischemic conditions (Figure 5) [42]. The upregulation of ASK1 in bladder ischemia and hypoxic smooth muscle cells is associated with morphological changes consistent with apoptotic degenerative responses, including cell shrinkage, fragmentation and condensation of the nucleus, partial disruption of the nuclear membrane, chromatin condensation, increased cytoplasmic and nuclear ribosomes, and the degradation of mitochondrial granules (Figure 5).

The precise mechanism by which ASK1 senses cellular stress and provokes stress responses remains largely elusive. Mediating factors are thought to involve c-June N-terminal kinase activity, the inhibition of calcineurine-nuclear factor of T-cells, and the instigation of Bax-mediated cell degeneration [54]. It was shown that the upregulation of ASK1 by ischemia contributes to the development of fibrosis, smooth muscle degeneration, and inflammatory responses [57]. Redox and the dithiol oxidoreductase binding partners mediate the degenerative actions of ASK1 in response to ischemia and redox through mechanisms involving p38 mitogen-activated protein kinases (p38α and β), c-June N-terminal kinases (JNK1, 2, and 3), and caspase-3 [57].

(3) Caspase-3: Activated caspase-3 senses the intensity of cellular stress and plays a leading role in the execution of apoptotic processes [42,58]. The upregulation of caspase-3 in bladder ischemia seems to be regulated by ASK1 [42]. ASK1 gene silencing by ASK1 siRNA in cultured bladder smooth muscle cells prevents the upregulation of caspase-3 by hypoxia [42]. It was shown that the activation of proteolytic and apoptotic activities by caspase-3 contributes to degenerative responses in brain ischemia [58]. The apoptotic action of caspase-3 under ischemic conditions appears to involve DNA damage and protein degradation [42,58]. The inhibition of caspase prevents ASK1-induced cell death, while the upregulation of ASK1 activates caspase-3 by stimulating mitochondrial cytochrome c [42,58]. These observations suggest that caspases may be required for cellular stress sensing and degenerative responses initiated by ASK1. The caspase-3 response paradigms in cellular stress are determined by the intensity of stressful stimuli [42,58]. In mild cellular stress, caspase-3 activates survival pathways to promote cell function and structural integrity [58]. However, severe cellular stress activates caspase-3 to initiate and execute apoptotic cell death [42]. It is thought that low caspase-3 activity in mild cellular stress activates cell survival signaling through mechanisms involving protein kinase B, also known as Akt [42,58]. The activation of Akt in low cellular stress was abolished by caspase-3 inhibitors and in caspase-3 knockout mice [58]. These observations imply the involvement of caspase-3 in modulating proper cellular response to stress when cells are exposed to unfavorable conditions, such as ischemia and redox.

The upregulation of the ASK1/caspase-3 pathway in bladder ischemia is associated with structural modifications involving the nucleus, nuclear membrane, and cytoplasmic organelles, consistent with apoptotic degenerative responses [42]. The upregulation of proapoptotic molecules, such as cytochrome c, in response to mitochondrial stress can activate caspase-3 and provoke the cleavage of Akt, an important regulator of antiapoptotic transcription factors [58]. This provokes the post-translational modification of cytochrome c-regulated cytosolic factors leading to the deterioration of subcellular elements and the loss of cellular structural integrity under ischemic conditions [42].

## 5. Signaling Pathways in Bladder Ischemia

Molecular signaling in bladder ischemia has not been fully defined from the perspective of the pathways developed after moderate and severe arterial insufficiency. The progressive phases of bladder ischemia are associated with varying changes depending on the severity and duration of ischemia and subsequent activation of cytokines, eicosanoids, leukotrienes, protein kinases, afferent signals, and cell survival responses. Downstream pathways involve signals from the membrane into the cytoplasm where kinases are activated and signal to the nucleus to activate the transcription factors involved in inflammatory and degenerative responses.

**Figure 6:**
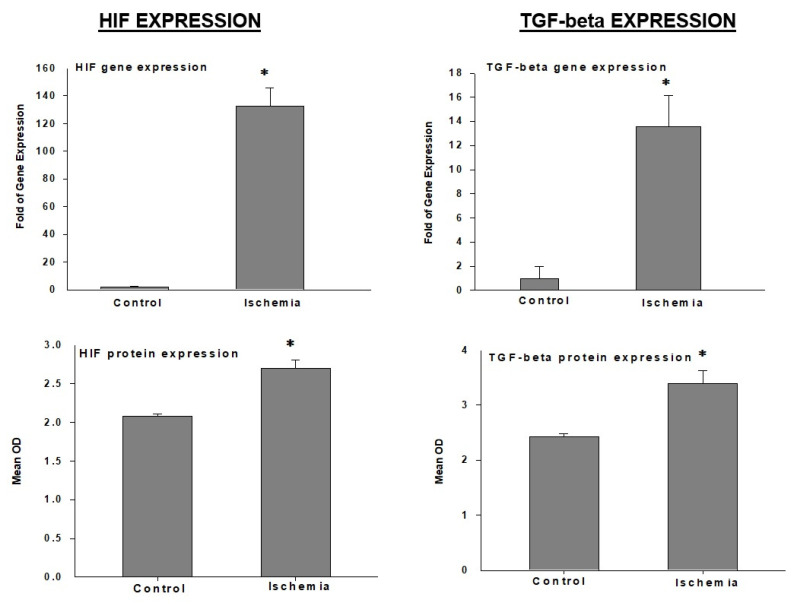
Expression of HIF and TGF-β in rabbit bladder tissues. * Indicates significant differences versus control. This figure is taken from our previous publication [6].

HIF: The hypoxia-inducible factor-1 (HIF-1) alpha gene and protein are significantly upregulated in bladder ischemia (Figure 6) [6]. HIF-1 is rapidly constrained and degraded under normoxia conditions [59,60,61,62]. In contrast, hypoxia upregulates HIF-1 and promotes its stabilization [59,60,61,62]. HIF-1 activates the transcription of a variety of genes that encode proteins involved in physiological directives regulating cell proliferation, energy metabolism, angiogenesis, cell cycle control, and cell death [6,62]. Increased expression of HIF-1 alpha in the ischemic bladder may suggest a tissue response to oxygen deprivation and redox [6,62].

The upregulation of HIF-1 has been documented in both bladder arterial insufficiency and bladder outlet obstruction [6,59]. Increased levels of the HIF-1 alpha gene and protein in bladder ischemia are associated with the loss of mitochondrial structural integrity and mitochondrial stress responses similar to those reported in free radical insult and oxidative stress conditions [6,59]. Mitochondrial stress responses in ischemia may contribute to the formation of free radicals and provoke oxidative stress in surrounding cellular components. It was shown that, under hypoxic conditions, HIF regulates the electrons passing through the mitochondrial electron transport chain and promotes their reaction with molecular oxygen [62]. This reaction produces superoxide, which intermingles with surrounding molecules and produces additional free radicals via enzymatic and non-enzymatic mechanisms [60]. Subsequent cellular episodes involve a cascade of degenerative responses mediated by the mitochondrial release of degenerating factors involving cytochrome c, endonuclease G, and apoptosis-regulating kinases to the cytosol [61]. These regulators activate cell danger signaling and lead to the disintegration of cellular organelles and activation of degenerative responses within cellular and subcellular structures [60,61].

TGF-beta: Among the transforming growth factor (TGF) superfamily, TGF-beta is abundantly expressed in the bladder and seems to be highly responsive to ischemia. The TGF-beta gene and protein are significantly upregulated in bladder ischemia (Figure 6) [6]. The upregulation of TGF-beta may contribute to structural modifications of smooth muscle cells, the deposition of connective tissue, the collagen invasion of nerve fibers, and the sporadic vacuolization of detrusor layers reported in bladder ischemia [6]. TGF-beta is an important regulator of extracellular matrix synthesis and degradation, playing a leading role in balancing the relative proportion of smooth muscle and connective tissue [4,6]. The deposition of collagen provoked by ischemia and mediated by TGF-beta has been shown to impair bladder compliance by compromising the fibroelastic properties of the detrusor tissue [4,6]. These observations in bladder ischemia are consistent with a clinical report of a close link between impaired bladder blood flow and non-compliance in elderly patients [30]. The ischemia-mediated upregulation of TGF-beta has also been documented in heart tissue and aortic smooth muscle cells [63]. It was shown that TGF-beta upregulates its own mRNA and increases its receptor expression under hypoxic conditions [63]. It is thought that fibroblasts, the main matrix producing cells, are the major target of TGF-beta and constitute the central effectors of connective tissue accumulation in fibrotic conditions [63]. In addition to its leading role in myofibroblast conversion, TGF-β exerts a wide range of regulatory effects on fibroblasts, including gene expression, survival, migration, and proliferation [63]. Because of its dominant role in regulating numerous pathways, TGF-β is recognized as an astounding molecule with perceptible effects on the remodeling of extracellular matrix. TGF-β may have a stimulating role in the gene transcription of structural collagens and may be implicated in the post-translational modification of collagen by enhancing its stability via cross-linking mechanisms [63].

**Figure 7:**
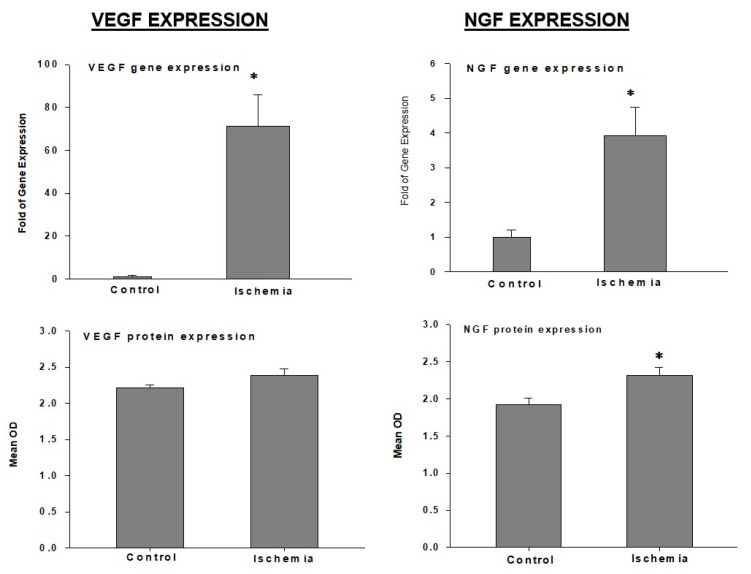
Expression of VEGF and NGF in rabbit bladder tissues. * Indicates significant differences versus control. This figure is taken from our previous publication [6].

VEGF: It was shown that bladder ischemia upregulates VEGF gene expression while having no significant effect on VEGF protein levels (Figure 7) [6]. The upregulation of the VEGF gene is thought to be a defensive response to invigorate angiogenesis, promote blood perfusion, and preserve microvasculature structural integrity in the ischemic bladder [6].

However, it seems that the upregulation of the VEGF gene in bladder ischemia fails to activate the downstream regulators required for VEGF protein production. Interruption of VEGF protein synthesis may be an important factor in the development of microvasculature endothelial damage, subintimal fibrosis, the disruption of endothelial cell junctions, the loss of vascular endothelial cells, and smooth muscle degeneration reported in the ischemic bladder [6,7]. The precise mechanisms by which ischemia impairs VEGF protein synthesis remain elusive. One possibility is that the ischemic tissues distressed by nutrient deficiency and low oxygen tension may not be capable of instigating VEGF protein synthesis. Other possibilities may relate to the accumulation of free radicals, interference with enzymatic activity, oxidative damage to the VEGF protein, and post-translational modifications of the VEGF protein. The upregulation of TGF-beta may also play a role in the suppression of VEGF protein synthesis in the ischemic bladder [64]. TGF-beta destabilizes the VEGF protein and reduces its expression through mechanisms involving ubiquitination and degradation [64]. In contrast, the upregulation of HIF under hypoxic conditions regulates the induction of VEGF transcription to promote angiogenesis [65]. Angiogenic growth factors, including VEGF, are required for the stimulation of angiogenesis to promote endothelial and smooth muscle cell migration under ischemic conditions [65]. VEGF functions as an essential survival factor for endothelial cells when oxygen levels decline and free radicals accumulate. It was shown that the suppression of VEGF synthesis adversely influences endothelial cells and leads to the detachment of endothelial cells from blood vessels [65].

NGF: Ischemia upregulates nerve growth factor (NGF) expression in the bladder at both transcriptional and translational levels (Figure 7) [6]. The upregulation of NGF may be an intrinsic defensive response against ischemia to promote neural outgrowth; preserve structural integrity of the nerve fibers; and prevent degenerative changes in the axon, Schwann cells, and myeline sheath in the bladder. However, NGF upregulation in bladder ischemia appears to fail to protect neural structural integrity because ischemia was shown to downregulate the p75 NGF receptor expression prior to upregulating NGF expression [66]. The rapid downregulation of the p75 NGF receptor by ischemia may be a primary factor in neurodegenerative responses and the containment of neural outgrowth in the ischemic bladder [66]. Impairment of the NGF/p75 NGF pathway in bladder ischemia may involve direct aspects of arterial insufficiency by means of hypoxia and nutrient meagerness or indirect belligerent factors involving free radical insult and the activation of deleterious oxidative interventions within the nerve fibers. The upregulation of NGF in non-ischemic conditions, such as diabetes, was shown to diminish sensory deficit, myelin degeneration, and impaired transmitter release from nerve endings [67]. The protective actions of NGF were shown to involve angiogenic activity and new microvascular outgrowth within Schwann cells and nerve fibers [67]. These observations suggest that the bladder responds to ischemia through well-coordinated intercommunications between HIF, VEGF, and NGF to initiate angiogenesis, improve perfusion, and provide the oxygen and nutrients needed for nerve fiber outgrowth and the protection of neural structural integrity [67]. However, prolonged ischemia and persisting free radical insult were shown to engender excessive oxidative products and provoke neurodegenerative responses by compromising the NGF/p75 NGF pathway and impairing NGF pro-survival signaling [67].

## 6. Differential Protein Expression and Post-Translational Protein Modifications in Bladder Ischemia

**Figure 8:**
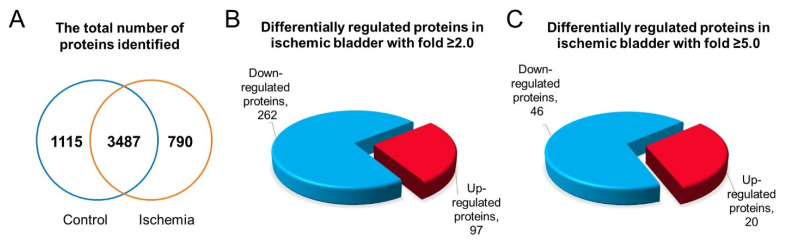
(**A**) Total number of proteins that are identified in the ischemic and control bladder tissue lysates. (**B**) Upregulated (≥2.0-fold) and downregulated (≥2.0-fold) proteins in the ischemic bladder. (**C**) Upregulated (≥5.0-fold) and downregulated (≥5.0-fold) proteins in the ischemic bladder. This figure is taken from our previous publication [48].

Ischemia compromises the bladder proteome by provoking differential protein expression and post-translational protein modifications [48,68]. Liquid chromatography–tandem mass spectrometry (LC-MS/MS) analysis identified 5392 proteins in the rat bladder (Figure 8A) [48] and revealed that ischemia significantly upregulates 172 proteins while downregulating 527 proteins in comparison with control bladder samples (*p* ≤ 0.05) [48]. When the differential expression of the proteins in bladder ischemia was assessed, considering at least two-fold increase or decrease, 97 upregulated and 262 downregulated proteins were detected in comparison with control samples (Figure 8B) [48]. When altered protein expression was assessed at five-fold increase or decrease, 20 upregulated and 46 downregulated proteins were detected in the ischemic bladder versus control (Figure 8C) [48].

**Figure 9:**
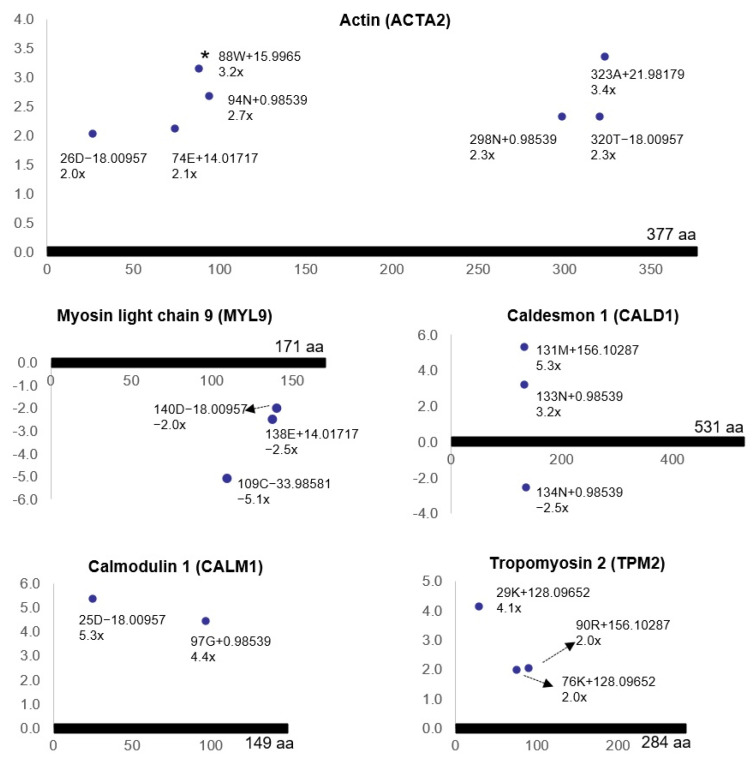
Post-translational modifications of contractile proteins (R^2^ > 0.5, ratio > 2, *p* < 0.05) in bladder ischemia. Dots represent non-coded amino acids (ncAAs) of each contractile protein. The annotation information of each dot is as follows: modified (protein) position, modified amino acid residue, delta mass, and ratio of modification. The horizontal axis represents the amino acid position of each protein; the vertical axis represents the ratio of modification (up or down). The asterisk denotes the oxidation of actin. This figure is taken from our previous publication [68].

Comparing the proteome of ischemic bladders with controls, 12 of the 23 amino acid variations were significantly dysregulated (R2 > 0.5, ratio > 2-fold, *p* < 0.05), suggesting the possibility of post-translational protein modifications [68]. Some of the ischemia-regulated amino acid variations that matched the molecular weights of amino acid substitution did not seem to be generated by point mutations at the DNA and/or RNA levels, suggesting the accumulation of non-coded amino acids (ncAAs) that might have resulted from post-translational modifications [68]. It was shown that bladder ischemia engenders post-translational modifications of the smooth muscle contractile proteins, including actin (ACTA2), myosin light chain 9 (MYL9), caldesmon 1 (CALD1), calmodulin 1 (CALM1), and tropomyosin 2 (TPM2) (Figure 9) [68].

In addition to contractile proteins, ischemia provokes post-translational modifications in a wide range of cellular stress response proteins in the bladder, including crystallin (CRYAB), heat shock protein 90 alpha family class B member 1 (HSP90AB1), heat shock protein 90 alpha class A member 1 (HSP90AA1), and heat shock protein family A (HSP70) member 8 (HSPA8) [68]. Others undergoing post-translational modifications in bladder ischemia are the proteins involved in the tetrahydrobiopterin (BH4) synthesis, recycling, salvage, and regulation pathway, including calmodulin 1 (CALM1), heat shock protein 90 alpha class A member 1 (HSP90AA1), and sepiapterin reductase (SPR) [68].

## 7. Gene Ontology, Pathway, and Network Analysis

**Figure 10:**
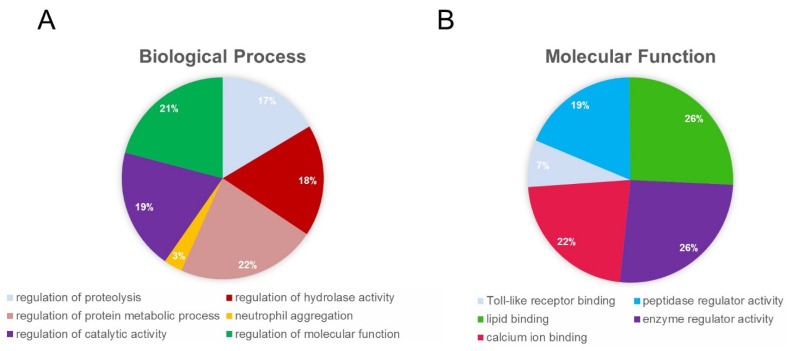
Gene ontology analysis of differentially expressed proteins in bladder ischemia. In this study, differentially expressed proteins with over 5-folds of changes were categorized using Gene Ontology (GO) bioinformatics tool. The GO terms with a *p*-value ≤ 0.05 were used to generate the pie charts. (**A**) Biological process and (**B**) molecular function. This figure is taken from our previous publication [48].

Biological and pathophysiological properties of altered proteins in bladder ischemia have been defined by gene ontology (GO), pathway, and network analysis [48,68]. These studies have revealed that differential protein expression and protein modifications provoked by ischemia contribute to a wide range of functional deficits and structural impairments in the bladder. Gene ontology (GO) analysis showed that 66 ischemia-regulated proteins (20 upregulated and 46 downregulated proteins with a greater than five-fold change) are involved in physiologic categories that regulate proteolysis, the protein metabolic process, and hydrolase activity based on biological process (Figure 10A) [48]. Based on molecular function, the 66 altered proteins are in the categories that regulate peptidase and other enzymatic activities (Figure 10B) [48]. These observations suggest that differential protein expressions in bladder ischemia contribute to a variety of pathophysiologic events by initiating peptidase activity, proteolysis, and protein degradation, leading to the dysfunction and loss of cellular structural integrity.

**Figure 11:**
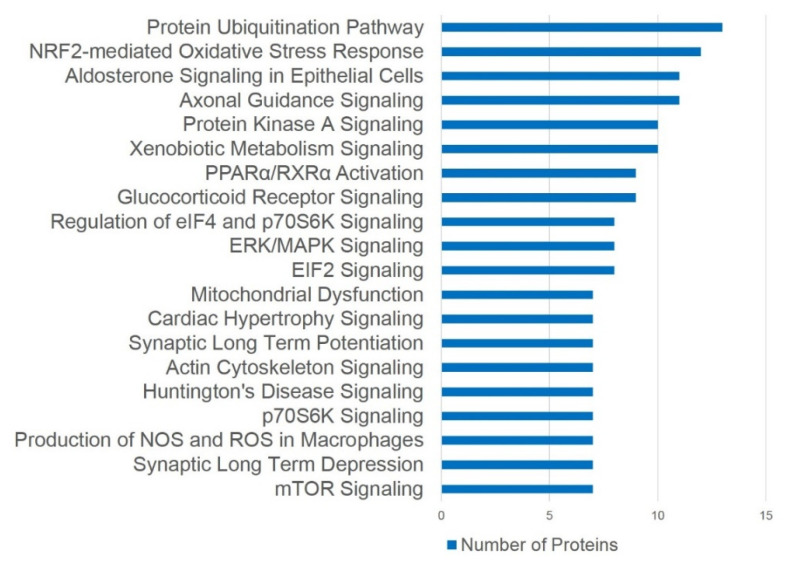
Canonical pathway analysis. The differentially expressed proteins (≥2.0-fold) in bladder ischemia are defined by pathway analysis using IPA software. Top 20 pathways are shown. This figure is taken from our previous publication [48].

An assessment of pathways using Ingenuity Pathways Analysis (IPA) software has suggested a close link between altered proteins and the activation of several signaling pathways involved in the structural deterioration of subcellular elements and degenerative responses in bladder ischemia (Figure 11) [48].

**Figure 12:**
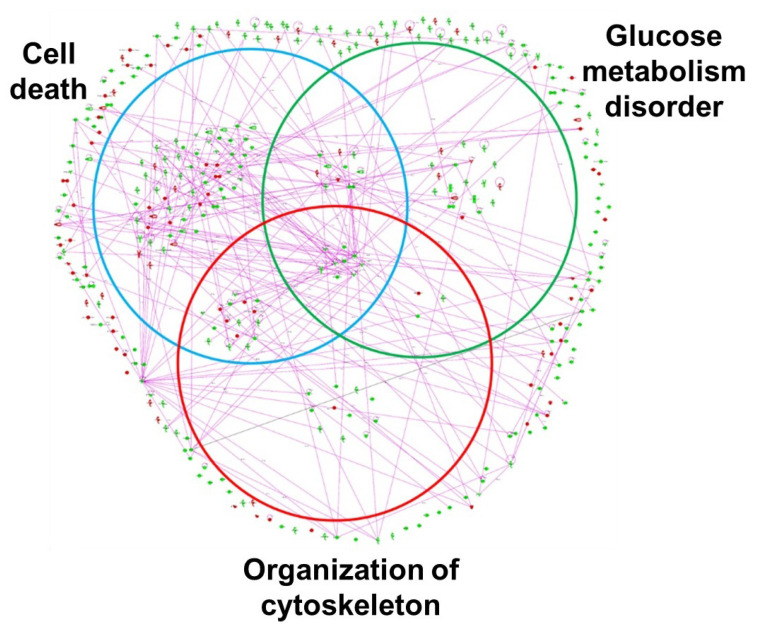
Network analysis of protein network in bladder ischemia by Ingenuity Pathways Analysis (IPA). Relevant networks were established from differentially expressed proteins (two-fold or greater) in ischemic group based on functional classification. Venn diagram networks were associated with subcategories related to cell death, glucose metabolism disorder, and cytoskeleton organization. Red dots indicate upregulated proteins and green indicate downregulated proteins. This figure is taken from our previous publication [48].

IPA canonical pathway analysis of 359 ischemia-regulated proteins (97 up- and 262 down-regulated at over two-fold of changes) has suggested that the highest number of altered proteins is associated with the ubiquitination pathway, followed by the nuclear factor-erythroid 2-related factor 2 (Nrf2)-mediated oxidative stress response (Figure 11) [48]. Altered proteins in bladder ischemia are also closely correlated with EIF2 signaling, ERK/MAPK signaling, actin cytoskeleton signaling, and mitochondrial dysfunction (Figure 11) [48]. Differentially expressed proteins in bladder ischemia appear to be involved in metabolic maladies, the disruption of cytoskeleton elements, and subcellular degenerative changes that ultimately lead to cell death (Figure 12) [48]. Network analysis with IPA has revealed that 134 of the 359 altered proteins are linked to structural damage and degenerative processes in bladder ischemia [48]. Among them, 98 proteins in the network are associated with subcategories related to cell death, 41 proteins are associated with glucose metabolism disorders, and 39 proteins are linked to cytoskeleton organization (Figure 12) [48].

**Figure 13:**
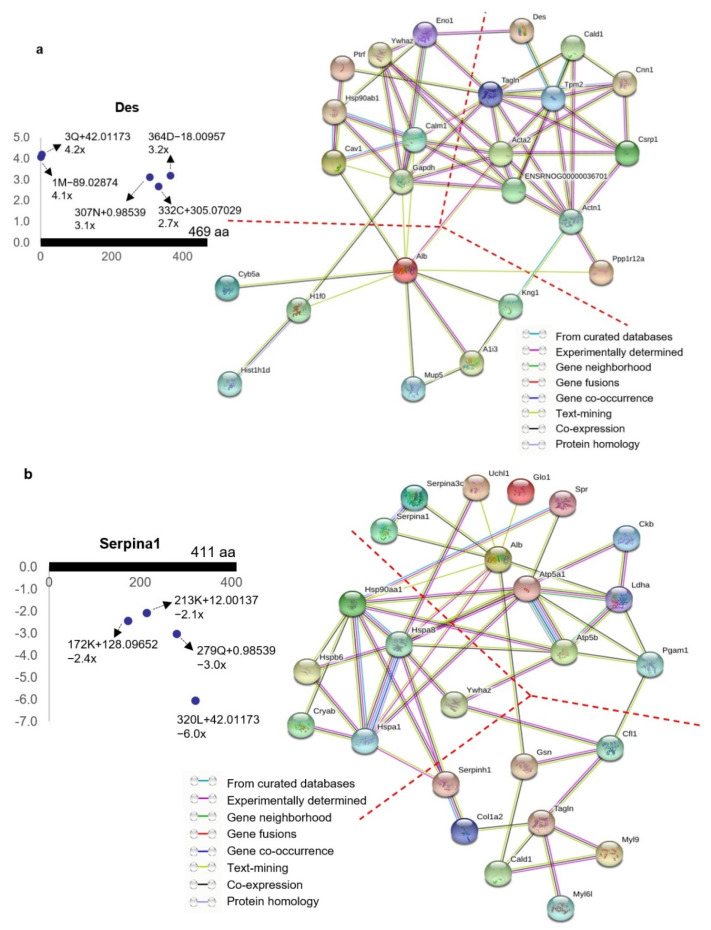
Protein–protein interaction networks of ischemia-associated ncAA-containing proteins. Network nodes and edges represent proteins and protein–protein associations, respectively. The color of the network edge indicates the type of interaction evidence. (**a**) Protein–protein interaction networks formed between 30 upregulated ischemia-associated ncAA-containing proteins (R^2^ > 0.5, ratio > 2, *p* < 0.05). Each protein had upregulated ischemia-associated ncAAs. Des is shown as an example. The annotation information of each dot, which represents ncAA, is as follows: modified (protein) position, modified amino acid residue, delta mass, and ratio of modification. The horizontal axis represents the amino acid position of Des; the vertical axis represents the ratio of modification. The interaction networks were broadly divided into three clusters: cell signaling-related proteins (**left**), cytoskeleton-associated proteins (**right**), and binding/transport-related proteins (**bottom**) (24 connected proteins are shown, and the clusters are divided with dotted lines). (**b**) Protein–protein interaction networks formed between 33 downregulated ischemia-associated ncAA-containing proteins (R^2^ > 0.5, ratio < −2, *p* < 0.05). Each protein had downregulated ischemia-associated ncAAs. Serpina1 is shown as an example. The annotation information of each dot, which represents ncAA, is as follows: modified (protein) position, modified amino acid residue, delta mass, and ratio of modification. The horizontal axis represents the amino acid position of Serpina1; the vertical axis represents the ratio of modification. The interaction networks were broadly divided into three clusters: molecular chaperones (**left**), metabolic enzymes (**right**), and cytoskeleton-associated proteins (**bottom**) (25 connected proteins are shown, and the clusters are divided with dotted lines). This figure is taken from our previous publication [68].

STRING (Search Tool for Recurring Instances of Neighbouring Genes) analysis of the ischemia-associated ncAA-containing proteins revealed that multiple interaction networks are formed between them [68]. The protein–protein interactions of the 30 upregulated ischemia-associated ncAA-containing proteins (R^2^ > 0.5, ratio > 2, *p* < 0.05) based on the STRING analysis showed that the interaction networks are broadly divided into three clusters: cell signaling-related proteins, cytoskeleton-associated proteins, and binding/transport-related proteins (Figure 13a) [68]. STRING analysis of the 33 downregulated ischemia-associated ncAA-containing proteins (R^2^ > 0.5, ratio < −2, *p* < 0.05) indicated that the interaction networks formed between them are mainly divided into three clusters: molecular chaperones, metabolic enzymes, and cytoskeleton-associated proteins (Figure 13b) [68]. Among the molecular chaperone cluster, stress-responsive heat shock proteins (HSPs) are predominant.

## 8. Conclusions

The notion of bladder ischemia as a contributing factor to LUTS is supported by epidemiological data and clinical observations suggesting a close link between vascular risk factors, pelvic ischemia, and the development of lower urinary tract dysfunction. Clinical studies imply that the same vascular risk factors leading to cardiovascular disease may provoke pelvic ischemia and engender LUTS in the elderly population. This concept is further supported by clinical reports suggesting that voiding dysfunction is more prevalent in patients with cardiovascular disease in comparison with same-age patients without cardiovascular disease. An improvement in bladder blood flow and the reversal of ischemia were shown to reduce LUTS in elderly patients. Ischemia compromises bladder structure and function by mechanisms involving oxidative radicals, oxidative stress-sensitive enzymes, cellular stress response molecules, the activation of cytokines, eicosanoids, leukotrienes, protein kinases, afferent signals, cell survival responses, and post-translational protein modifications. These changes in bladder ischemia could provide valuable diagnostic targets and lead to potential therapeutic strategies against LUTS in patients with non-obstructed bladder. From a clinical standpoint, it is critical to develop medical devices for a non-invasive measurement of human bladder blood flow and a reliable diagnosis of bladder ischemia. Medical co-morbidities should be identified by obtaining a careful history and treated in conjunction with LUTS. Elderly patients with LUTS may require medications to treat their symptoms as well as therapeutic strategies to prevent oxidative stress and protect the bladder tissue against ischemic damage. Further research into the pathophysiology of lower urinary tract ischemia may lead to a more effective management of LUTS in elderly patients.
